# Separate vertical wiring plus bilateral anchor girdle suturing fixation for the fractures of the inferior pole of the patella

**DOI:** 10.1186/s13018-023-03649-0

**Published:** 2023-03-08

**Authors:** Shi-Jie Li, Shashi Ranjan Tiwari, Shi-Min Chang, Shou-Chao Du, Ying-Qi Zhang

**Affiliations:** 1 Department of Orthopaedic Surgery, Yangpu Hospital, School of Medicine, Tongji University, 450 Tengyue Road, Shanghai, 200090 China; 2 Department of Orthopedic Surgery, Tongji Hospital, Tongji University School of Medicine, Shanghai, China

**Keywords:** Anterior tension band wiring, Separate vertical wiring, Bilateral anchor girdle suturing, Inferior pole fractures, Patella

## Abstract

**Background:**

The fixation of inferior pole fractures of the patella (IPFPs) is still a great challenge for surgeons.

**Materials and methods:**

We introduced a new fixation method for IPFP fixation, that is, separate vertical wiring plus bilateral anchor girdle suturing fixation (SVW-BSAG). Three finite element models including the anterior tension band wiring (ATBW) model, separate vertical wiring (SVW) model and SVW-BSAG model, were built to evaluate the fixation strength of different fixation methods. A total of 41 consecutive patients with IPFP injury were enrolled in this retrospective study, including 23 patients in the ATBW group and 18 patients in the SVW-BSAG group. The operation time, radiation exposure, full weight-bearing time, Bostman score, extension lag versus contralateral healthy leg, Insall–Salvati ratio, and radiograph outcomes were employed to assess and compare the ATBW group and SVW-BSAG group.

**Results:**

The finite element analysis confirmed that the SVW-BSAG fixation method was as reliable as the ATBW fixation method in terms of fixed strength. Through retrospective analysis, we found that there was no significant difference between the SVW-BSAG and ATBW groups in age, sex, BMI, fracture side, fracture type, or follow-up time. There were no significant differences between the two groups in the Insall–Salvati ratio, 6-month Bostman score, and fixation failure. Compared with the ATBW group, the SVW-BSAG group showed advantages in intraoperative radiation exposure, full weight-bearing time, and extension lag versus the contralateral healthy leg.

**Conclusion:**

The finite element analysis and clinical results showed that SVW-BSAG fixation methods are a reliable and valuable for IPFP treatment.

**Supplementary Information:**

The online version contains supplementary material available at 10.1186/s13018-023-03649-0.

## Introduction

Patellar inferior pole fracture refers to a fracture of the lower quarter of the patella, which either does not involve or rarely involves the articular surface of the patella. Due to the high rates of pain, nonunion, and permanent disability, the outcomes of non-operative treatment are not satisfactory in patellar inferior pole fractures. Therefore, these fractures are typically managed operatively.

Tension band techniques are the most common and reliable methods for the fixation of displaced patellar fractures [1–3]. However, inferior pole fractures of the patella (IPFPs) are often difficult to fix by tension band wiring due to their small bone fragments and comminution, especially in older patients with osteoporosis [4, 5]. Many surgical techniques have been proposed for the restoration of the extensor mechanism in IPFPs, including circumferential wiring [6, 7], a basket plate [8], a suture anchor with Krackow-Bunnell weave [9], and separate vertical wiring [10, 11]. However, all of these techniques have their own limitations.

In this paper, we present the surgical technique of separate vertical wiring (SVW) through cannulated screws combined with bilateral suture anchor girdle (BSAG) fixation in IPFPs. Moreover, we compared the biomechanical and clinical outcomes of anterior tension band wiring (ATBW) fixation and SVW-BSAG fixation techniques. It has been proven that the SVW-BSAG technique is a simple and reliable method for IPFP fixation.

## Material and methods

### Study population

A retrospective cohort study was performed using data derived from the Shanghai Yangpu Hospital and Tongji Hospital databases after being reviewed and approved by the ethics committee of the hospital (LL-2021-WSJ-007). IPFP-injured patients aged 18 years or older who underwent operative treatment in these two hospitals from January 2016 to December 2021 were included in this study. With economic conditions permitting, patients were randomly assigned to the ATBW group or the SVW-BSAG group. Due to financial difficulties, three patients undergoing fixation with the Krackow-Bunnell weave technique were excluded. Patients with open fractures or concomitant fractures (*n* = 2), those who were lost to follow-up at 6 months (*n* = 3), those who died within 6 months postoperatively (*n* = 2) and those with previous fracture of the ipsilateral knee (*n* = 1) were also excluded. After applying the inclusion and exclusion criteria, 41 consecutive patients with IPFP injury were enrolled in this study, including 23 patients with ATBW fixation (24 to 82 years of age) and 18 patients with SVW-BSAG fixation (26–85 years of age). The demographic information of patients is shown in the Supplement.

### Operative techniques and postoperative rehabilitation

Patients were placed supine on the operating table. After anesthesia, routine disinfection and preparation of towels were performed. The midline skin incision approach of the patella was used in all patients to expose the whole patella. After eliminating the clots and periosteum in the fracture gap, reduction in fragments was performed under direct vision.

In the ATBW group, two guide pins were inserted in parallel into the patella and perpendicularly across the fracture. The position of the guide pin was adjusted according to the anteroposterior and lateral C-arm radiographs until the position of the pin was satisfactory. Cannulated screws were inserted along with the guide pin, and then a cable or wire was fixed as a figure-of-8 tension band through the cannulated screws.

**Fig. 1:**
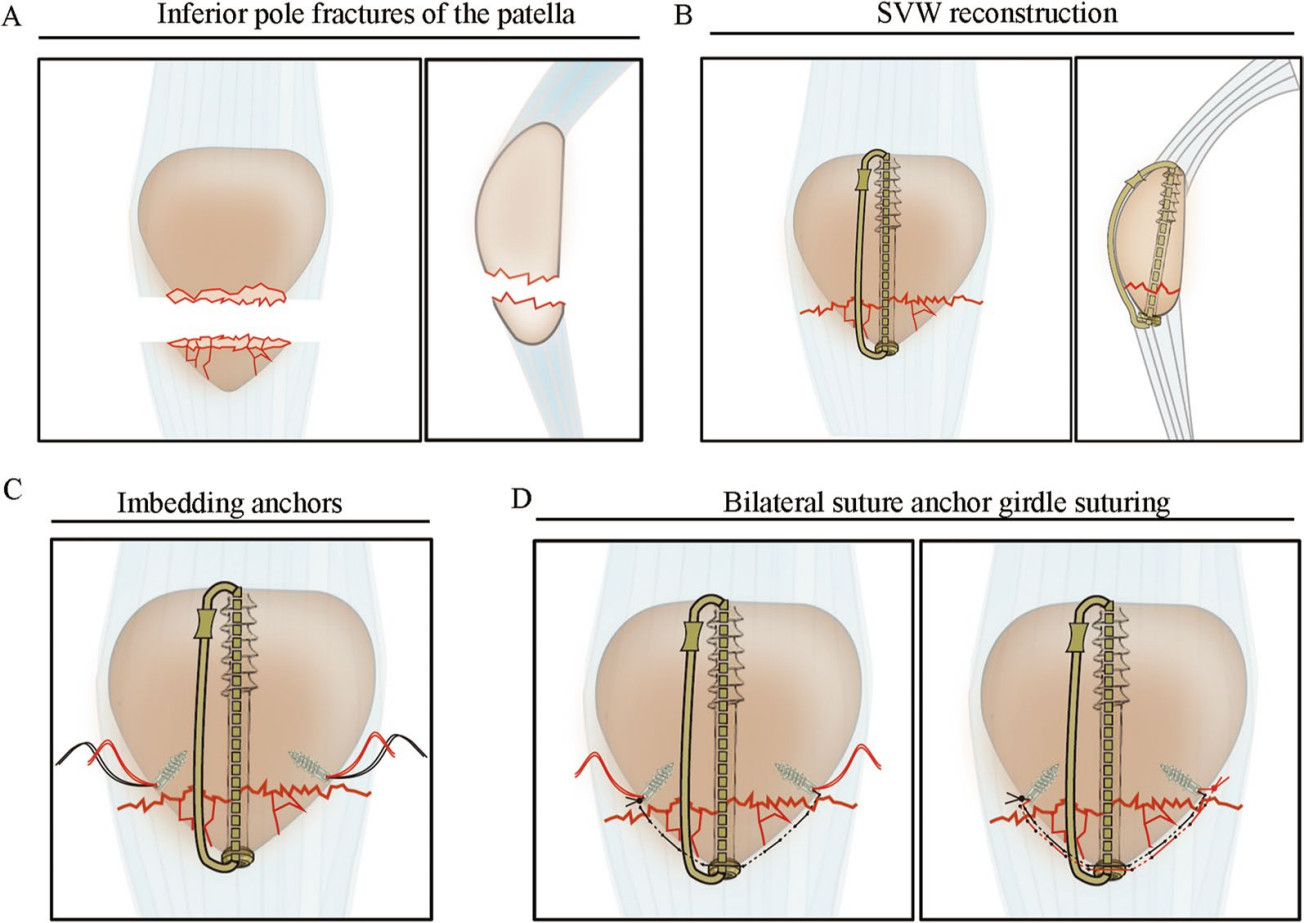
The operation diagram of SVW-BSAG fixation in the IPFP. **A** AP view and lateral view of the IPFP. **B** The IPFP reduction was performed, and an SVW technique through cannulated screws and cables was used to fix the fracture. **C** Two suture anchors were imbedded in the proximal fragment on both sides of the patella. **D** Separate vertical wiring plus bilateral anchor girdle suturing fixation

In the SVW-BSAG group, a single guide pin was inserted into the patella along the long axis of the patella and perpendicularly across the fracture. The position of the guide pin was adjusted according to the anteroposterior and lateral C-arm radiographs until the position of the pin was satisfactory. After a cannulated screw was inserted along the guide pin, a cable was separated by vertical wiring through the cannulated screw (Fig. 1A–B).

Two suture anchors were placed into both sides of the proximal fragment (Fig. 1C). The entry portal of the anchor was at least 1 cm proximal to the transverse fracture line on the coronal plane and approximately 5 mm posterior to the anterior cortical surface on the cross section. Then, the anchor suture string was sutured along the inferior border of distal fragments on the patellar tendons and knotted with one of the contralateral anchor lines at the entry portal of the contralateral anchor (Fig. 1D). The same procedure was repeated for the remaining three anchor lines (Fig. 1D).

In the two groups, patients were required to wear a hinged knee brace for 2 weeks postoperatively. For the first 2 weeks postoperatively, the hinged knee brace was locked in full extension during weight bearing and unlocked when patients were to perform flexion and extension of the knee without weight bearing. Weight-bearing knee flexion and extension exercises were allowed 2 weeks after the operation. Normal passive full-range motion of the knee was required at 4 weeks postoperatively.

### Postoperative evaluation

Patient baseline data, including sex, age, fracture side, and body mass index (BMI), were extracted from the hospital database. The operation time was defined as the time from the moment of incision to the time of incision closure. Radiation exposure was assessed by the radiation dose gathered from the C-arm fluoroscopy machine (GE Healthcare, USA) postoperatively. Full weight-bearing time was defined as the time when patients could walk without any assistive device or only with a walking stick to maintain balance postoperatively.

All patients were followed for at least 6 months. A Bostman score [15] and extension lag versus the contralateral healthy leg were employed to assess the postoperative knee function of the IPFP patients at 6 months postoperatively. A Bostman score of 28 or higher was considered excellent in regard to the functional recovery of the knee, 20–27 was considered good, and less than 20 was considered poor. The Insall–Salvati ratio [16] was assessed on the immediate postoperative radiograph. The radiograph outcomes were evaluated by anteroposterior and lateral radiographs at 1 and 3 months postoperatively.

### Statistical analysis

Statistical analyses were conducted using SPSS software (SPSS 22.0, SPSS Inc.). Continuous variables are expressed as the mean ± standard deviation, whereas categorical variables are expressed as counts. For continuous variables with a normal distribution, Student’s t test was used to evaluate the difference between the two groups. For continuous variables with a non-normal distribution, the Mann–Whitney U test was employed. Categorical variable associations were examined using the chi-square test. *p* values less than 0.05 were considered statistically significant.

### Finite element study

#### Finite element models

The three-dimensional finite element knee model including the patella, the tendon of the patella, and the distal femur was reconstructed from the CT data of the right knee of a 50-year-old female subject. The IPFP model was created by transecting the patella model at the distal pole of the patella. Then, standard surgical techniques were simulated to instrument the ATBW, SVW, and SVW-BSAG fixation constructs into the IPFP models, which were built to simulate and compare the biomechanical characteristics of the three fixation types.

Two cannulated screws (3 mm in diameter) and a cable (2 mm in diameter) were applied to form the ATBW model, which is a modified tension band fixation (Additional file 1: Figure S1A). The SVW model is built with a cannulated screw (3 mm in diameter) and a cable (2 mm in diameter). In this model, the IPFP injury was only fixed by separate vertical wiring (Additional file 1: Figure S1B). The SVW-BSAG model is built with a cannulated screw (3 mm in diameter), a cable (2 mm in diameter), and two suture anchors (2 mm in diameter). In this model, the cannulated screw and the cable were implanted as in the SVW model. The suture anchor was angled 45 degrees below the horizontal with implantation into the proximal patellar fragment, and the anchor lines were simplified into an irregular cylinder with a diameter of 1 mm fixed on bilateral anchors and the patellar tendon (Additional file 1: Figure S1C). The elements and nodes of the three models are shown in Additional file 1: Table S1.

#### Finite element analysis

The femur was identified as a rigid body. The other models and implants were identified as linear elastic isotropic materials. The elastic moduli of the patella, patellar tendon, cannulated screw, suture anchor and anchor line were set as 15 GPa, 660 MPa, 110 GPa, 110 GPa, and 200 MPa, respectively, according to material properties and practices [12–14]. The Poisson's ratio of the patella, cannulated screw, and suture anchor was 0.3. The Poisson's ratios of the patellar tendon and anchor line were 0.4 and 0.36, respectively (Table 1). The friction coefficient at the anchor-bone interface was 0.6. Starting with a preload of 30 N, the anchoring system was strained.

**Table 1:** The Properties of the materials in the biomechanical test

	Patella	Cartilage	Patellar tendon	Cannulated screw	Cable	Suture Anchor	Anchor line	Femur
Specification	–	Thickness:3 mm	–	diameter:3.0 mm	Diameter: 2 mm	diameter: 1 mm	diameter:0.5 mm	–
Elastic modulus	15GPa	5 MPa	660 Mpa	110GPa	110GPa	110GPa	200 MPa	Rigid
Poisson's radio	0.3	0.46	0.4	0.3	0.3	0.3	0.36	

The change in the fracture gap and stress distribution on the components of the models were employed to evaluate the stability of different fixation types. Three paired points were defined on the proximal fragment and distal fragment of the patella to evaluate the displacement of fragments after loading. All of the finite element analyses were completed in ANSYS 17.0.

## Results

### Finite element analysis results

**Fig. 2:**
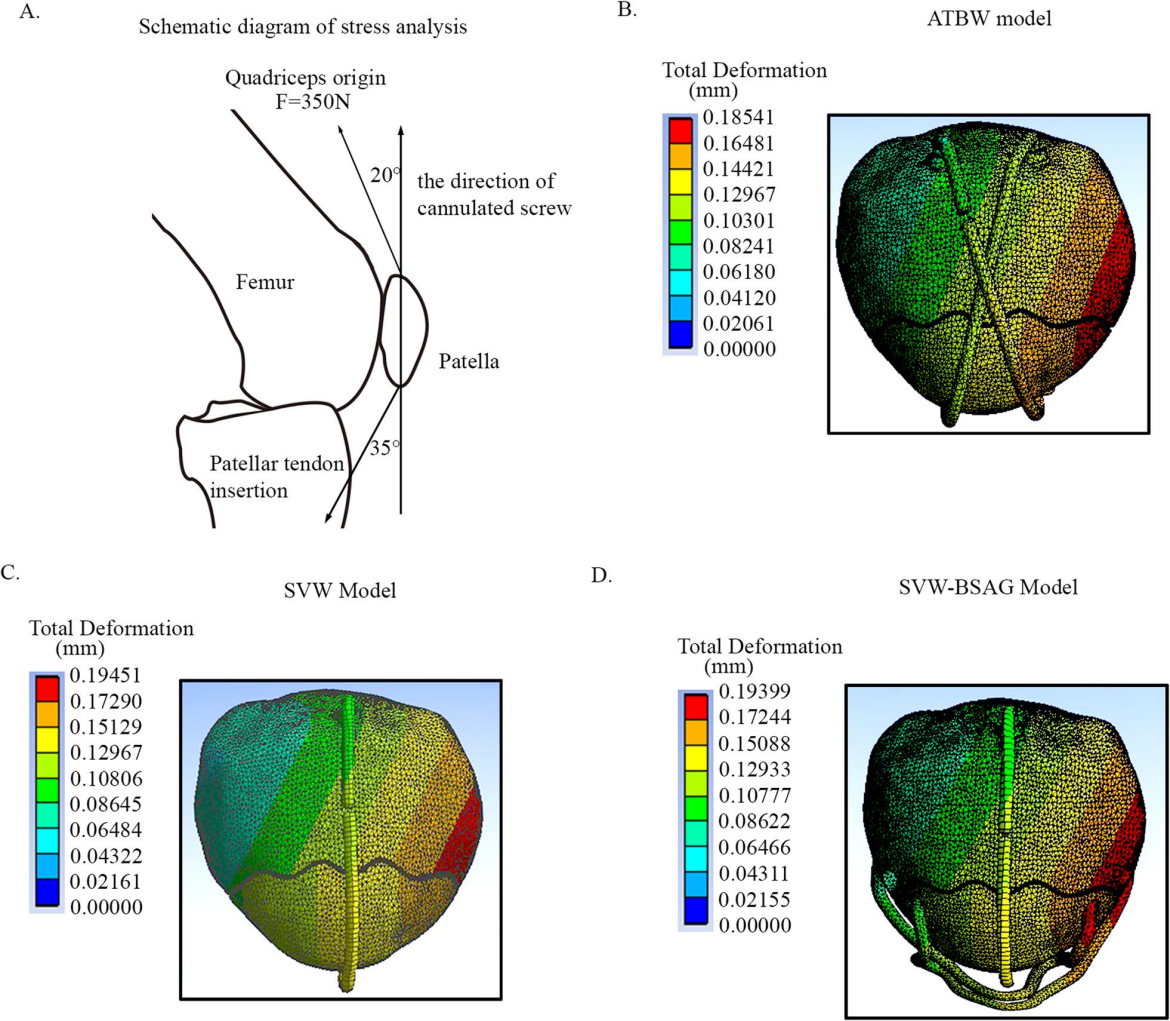
Finite element analysis and stress displacement nephograms of the models. **A** The loading method of the IPFP finite element analysis. **B** The stress displacement of the ATBW model. **C** The stress displacement of the SVW model. **D** The stress displacement of the SVW-BSAG model

Based on previous biomechanical studies, the load state of the finite element simulation has been determined [17–19]. The model was set at a 45-degree flexed position of the knee joint, and the articular surface of the patella was in contact with the femur. The contraction direction of the quadriceps femoris ligament was angled 20 degrees with the long axis of the patella on the lateral view. The patellar tendon was angled 35 degrees with the long axis of the patella, and the distal end of the patellar tendon was considered fixed. A load of 350 N was applied to the proximal patellar fragment in the quadriceps femoris tendon contraction direction (Fig. 2A).

After finite element analysis of the three models, the fracture gap was acquired from 3 pairs of points in each model. The displacement of the fracture gap after loading in the ATBW model was 0.03–0.07 mm, which is in line with previous studies and indicates that the model methods in this study are credible and comparable (Fig. 2B). The displacements of the 3 pair points on the fracture gap in the SVW model were 0.09, 0.16, and 0.11 mm (Fig. 2C). The displacements of the 3 pair points on the fracture gap in the SVW-BSAG model were similar to those in the ATBW model, which were 0.04, 0.07, and 0.06, respectively (Fig. 2D, Table 2).

**Table 2:** The maximum stress and the same point relative displacement of the three models

Paraments		ATBW Model	SVW Model	SVW-BSAG Model
Same point displacement (mm)	Left	0.03	0.09	0.04
	Median	0.07	0.16	0.07
	Right	0.05	0.11	0.06
Maximum stress (MPa)	Patella	16.0576	37.0758	17.2842
	Internal fixation	50.306	87.383	54.521

After loading, the maximum stress of the patella in the SVW-BASG model was 17.2842 MPa, which is far less than the 50 MPa yield strength threshold of cortical bone. The maximum stress of internal fixation in the SVW-BSAG model was 54.521 MPa, which is less than the 827 MPa yield stress of titanium alloy. These results indicated that SVW-BSAG fixation is a reliable fixation method for IPFP injury (Table 2).

### Clinical outcomes

**Fig. 3:**
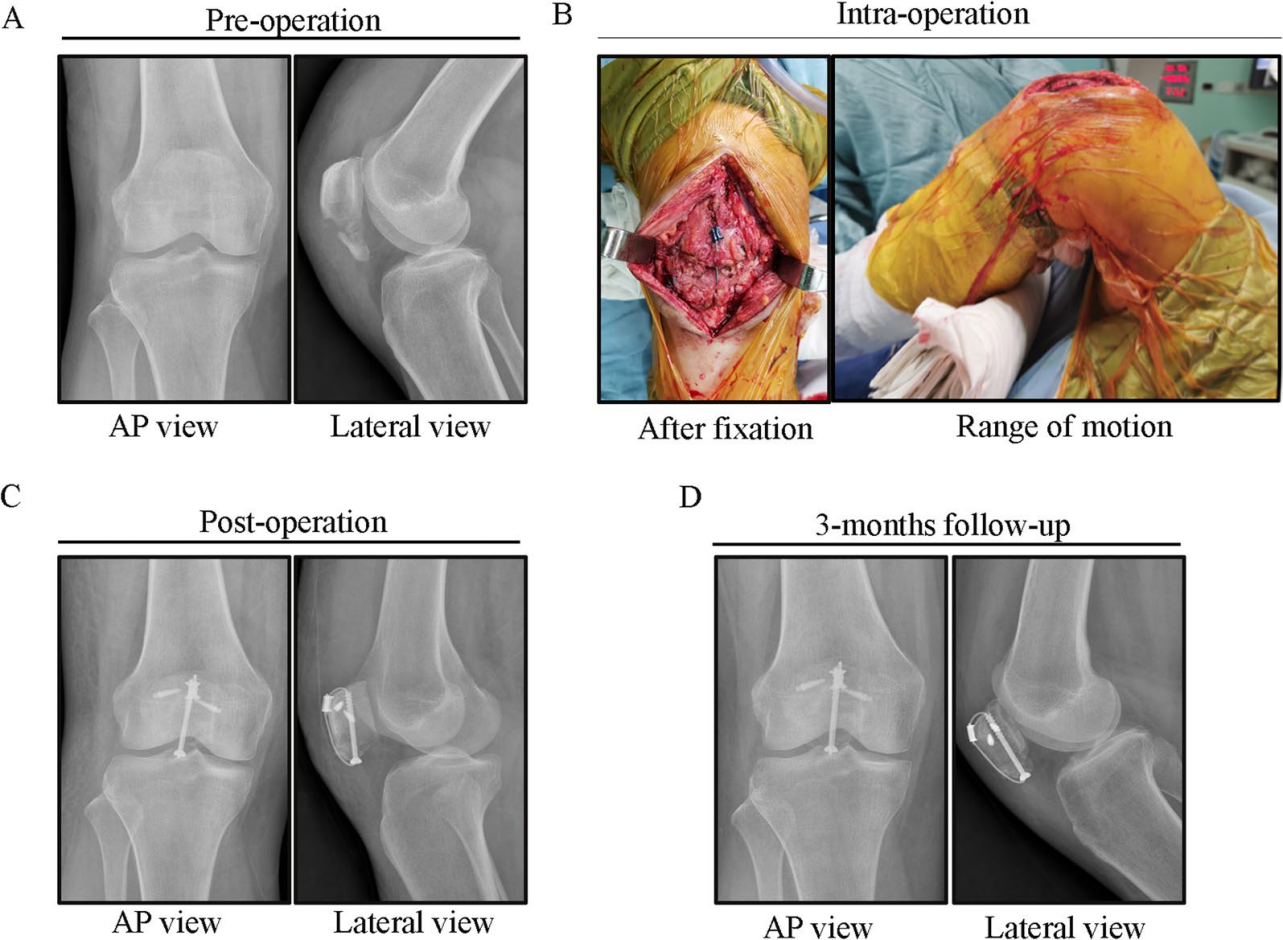
A typical case of an SVW-BSAG fixation IPFP patient. **A** This was a 47-year-old male patient with IPFP. **B** The patient underwent reconstruction of the IPFP through SVW-BSAG fixation. **C** Postoperative radiograph showed that the fracture was fixed in a good position. **D** After a 3-month follow-up, the patellar fracture healed well, and the injured knee had full range of motion

A typical case of the SVW-BSAG group is shown in Fig. 3. A typical case of the ATBW group is shown in Additional file 1: Figure S2. There were no significant differences between the ATBW group and the SVW-BSAG group with regard to age, sex, BMI, fracture side, fracture type, follow-up time, and operation times. The baseline and operation data are shown in Table 3. The radiation exposure in the ATBW group was 8.66 ± 2.27 cGycm^2^ versus 4.80 ± 1.81 cGycm^2^ in the SVW-BSAG group. The difference in radiation exposure between the two groups was significant (*p* < 0.0001). This may be due to one more cannulated screw needing to be placed satisfactorily under fluoroscopy in the ATBW group than in the SVW-BSAG group. The full weight-bearing times in the ATBW group and the SVW-BSAG group were 5.65 ± 1.27 weeks and 4.34 ± 1.20 weeks, respectively. Compared with the ATBW group, the full weight-bearing times in the SVW-BSAG group were much earlier (*p* = 0.005). The average Insall–Salvati ratio in the SVW-BSAG group was 0.97 ± 0.06 compared to 0.96 ± 0.05 in the ATBW group. There was no significant difference in the Insall–Salvati ratio between the two groups (*p* = 0.904).

**Table 3:** Clinical outcomes of patients

Paraments	SVW-BSAG Group	ATBW group	P-value
*Age*	52.22 ± 17.81	54.39 ± 15.71	0.681*
Sex (man/women)	8/10	13/10	0.443^§^
Fracture side (left/right)	11/7	13/10	0.767^§^
BMI	23.42 ± 2.32	23.32 ± 2.12	0.885*
*Fracture type*			0.775^§^
Communited	14	17	
Single	4	6	
Operation time (minutes)	55.06 ± 7.73	60.74 ± 10.59	0.063*
Radiation exposure (cGycm2)	4.80 ± 1.81	8.66 ± 2.27	0.000*
Bostman score (6 months)	27.00 ± 3.09	26.09 ± 3.33	0.294^†^
*Knee function*			0.576^§^
Excellent	9	8	
Good	8	14	
Poor	1	1	
*Full weight-bearing time (weeks)*	4.39 ± 1.20	5.65 ± 1.27	0.005*
Extension lag	3.33 ± 5.94	8.70 ± 9.44	0.061^†^
Fixation failure	1	1	0.859^§^
Insall–Salvati ratio	0.97 ± 0.06	0.96 ± 0.05	0.638*
Fracture union time (months)	4.05 ± 1.21	4.48 ± 1.22	0.175^†^
Follow-up time	8.78 ± 2.26	8.7 ± 2.08	0.904*

*Two-independent-sample t-test

^§^Chi-squared test

^†^Mann–Whitney U test

Both groups had a patient with implant failure, and both individuals underwent reoperation. A patient in the ATBW group was observed to have redisplacement of the distal fragments at the 1-month follow-up. This may have been due to an improper technique used during surgery, which resulted in further destruction of the distal pole fragments and secondary fixation failure (Additional file 1: Figure S2A). The patient received a reoperation by separate vertical wiring through cannulated screw augmented Krackow-Bunnell weave fixation. A patient in the SVW-BSAG group was observed to have implant failure at the 1-month follow-up (Additional file 1: Figure S2B). This may have been due to excessive weight-bearing exercise. The patient received reoperation by pole section.

All cases in both groups achieved bony union. The mean fracture union time was 4.05 ± 1.21 months in the SVW-BSAG group versus 4.48 ± 1.22 months in the ATBW group. There was no significant difference in terms of fracture union time between the two groups (*p* = 0.175). There were no significant differences between the two groups in the 6-month Bostman score (*p* = 0.234) or knee function recovery (*p* = 0.576). The average Bostman score was 27.00 ± 3.09 in the SVW-BSAG group versus 26.09 ± 3.33 in the ATBW group. Knee function recovery was excellent in 8 patients, good in 14 patients, and poor in 1 patient in the ATBW group. The outcome was excellent in 9 patients, good in 8 patients, and poor in 1 patient in the SVW-BSAG group. Most of the patients in the SVW-BSAG group achieved full knee extension. Five patients in the SVW-BSAG group had terminal flexion loss versus 12 patients in the ATBW group. There was no significant difference between the two groups in terms of the average extension lag, which was 3.33 ± 5.94 degrees in the SVW-BSAG group and 8.70 ± 9.44 degrees in the ATBW group (*p* = 0.061).

## Discussion

IPFP, which accounts for approximately 5% of patellar fractures, either does not involve or rarely involves the articular surface of the patella. However, disruption of the extensor mechanism always occurs in this injury and requires surgical treatment. Tension band wiring is a typical fixation technique for patellar fractures, that can transverse the tensile forces generated from the extensor mechanism into compressive forces on both sides of the fractured end to achieve stability. Our team also achieved good outcomes in the treatment of IPFP with ATBW through cannulated screws [20]. However, the distal fragments in IPFP are usually composed of a larger fragment located at the lowest aspect of the long axis of the patella and smaller comminuted fragments on both sides inferiorly [21]. Simple tension band wiring is difficult to perform in these injury cases and is limited to fixating the larger fragment at the lowest of the long axis of the patella. Insufficient fixation of comminuted fragments limits early functional exercise of patients postoperatively and may lead to the loss of range of motion of the knee [22]. In our study, patients in the ATBW group also required immobilization for 2 weeks.

This complex fracture precludes construction, and partial patellectomy is considered in some cases of IPFP. However, patella baja significantly restricts the range of motion of the knee and is subject to traumatic arthritis. Meanwhile, the difficult healing of the bone–tendon interface requires a longer immobilization time. Restoration of the patella has become the consensus of IPFP internal fixation surgery. Kastelec et al. introduced a basket plate to reconstruct the IPFP, which could construct the patella height and allow immediate mobilization and early weight-bearing [8, 22]. However, tendon and soft tissue irritation is a worrisome issue for plates in IPFP [23–25]. Similar concerns exist for rim plate technology [26].

A suture anchor was considered a reliable technique for IPFP reconstruction. A biomechanical study confirmed that the suture anchor constructed IPFP model can cyclic load 100 N of force and has less gapping [27]. Kim et al. used a novel suture bridge anchor fixation technique to achieve patellar height reconstruction, no postoperative patella-femur pain, and no limited range of motion in comminuted IPFP. Swensen et al. also reported a successful suture fixation case for IPFP. However, the strength of suture anchor fixation is limited. Patients are required to wear a knee immobilizer for 4–6 weeks, which precludes early functional exercise.

Yang et al. introduced separate vertical wiring for the fixation of comminuted IPFP and achieved satisfactory outcomes. However, the ultimate load of separate vertical wiring is only 250 N according to a cadaveric IPFP model biomechanical analysis [11]. Moreover, traditional separate vertical wiring uses three 0.75 mm diameter wires directly fastened to the patella. The tightening of steel wires makes it very easy to further cut the comminuted fractures after loading. Thus, separate vertical wiring does not allow patients to engage in early functional exercise either.

Therefore, we modified the separate vertical wiring by using a cable and cannulated screw, which could immobilize the main fragment in the IPFP. The finite element analysis confirmed that the SVW model can withstand 350 N loading with a tiny displacement of the fracture gap. Furthermore, bilateral suture anchor girdle fixation was employed to strengthen the fixation of the comminuted distal pole in the IPFP. We believe that this suture augmentation method initiated by our team can simply and effectively fix comminuted distal pole fragments, which is an effective supplement to separate vertical wiring technology. According to the finite element analysis, the overall stability of the SVW-BSAG model is similar to that of the ATBW model and can withstand a tensile force of 350 N. The force loaded to the completely extended quadriceps muscle is less than 316 N. Therefore, the SVW-BSAG model not only has a good effect on reduction but, theoretically, also allows patients to begin early functional exercise. To simplify the model and facilitate analysis, the finite element model for the inferior pole patella fracture was set as a simple fracture. The result of finite element analysis can only demonstrate the simple fracture pattern, which is a limitation of this study.

According to our research, the range of knee flexion loss in the SVW-BSAG group was significantly smaller than that in the ATBW group. The full weight-bearing times were also earlier in the SVW-BSAG group. This may have been because the IPFP patients receiving SVW-BSAG fixation engaged in immediate postoperative functional exercises. Notably, surgeons can more easily perform SVW-BSAG fixation than ATBW fixation in comminuted IPFP, as the former does not require the arrangement of double cannulated screws in the comminuted distal pole and thus does not cause concern regarding too many screws causing further damage to the inferior fragments. Therefore, it is reasonable that the SVW-BSAG group has an advantage in terms of radiation exposure.

## Conclusion

In conclusion, the SVW-BSAG technique exhibited good biomechanical results in simple inferior pole patella fracture model, as well as satisfying functional outcomes and less radiation exposure in clinical practice. We believe that the SVW-BSAG technique can provide stable fixation and allow early functional exercise, making it a simple and effective fixation procedure for IPFP patients and surgeons.

## Supplementary Information

**Additional file 1. **Supplemental Figures and Table.

## Data Availability

The datasets used and/or analyzed during the current study are available from the corresponding author on reasonable request.
