# Supplementary material for: Separate vertical wiring plus bilateral anchor girdle suturing fixation for the fractures of the inferior pole of the patella
Source: J Orthop Surg Res. 2023 Mar 8;18:176. doi: 10.1186/s13018-023-03649-0 (PMC9997027; doi:10.1186/s13018-023-03649-0)
Supplement: Supplementary file 1 — Additional file 1. Supplemental Figures and Table. [file 13018_2023_3649_MOESM1_ESM.docx]

**Supplement**

Figure S1


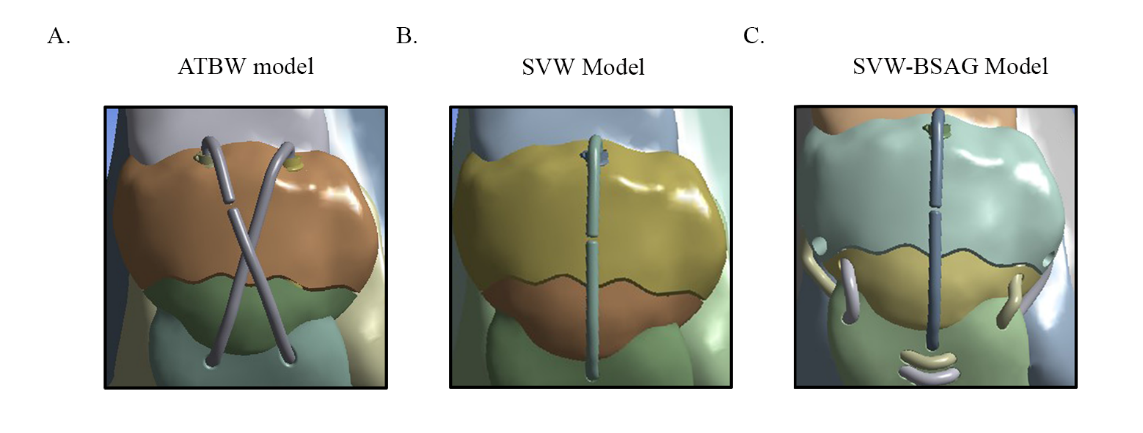


Figure S1. The fixation models in the ANSYS.

Figure S2.


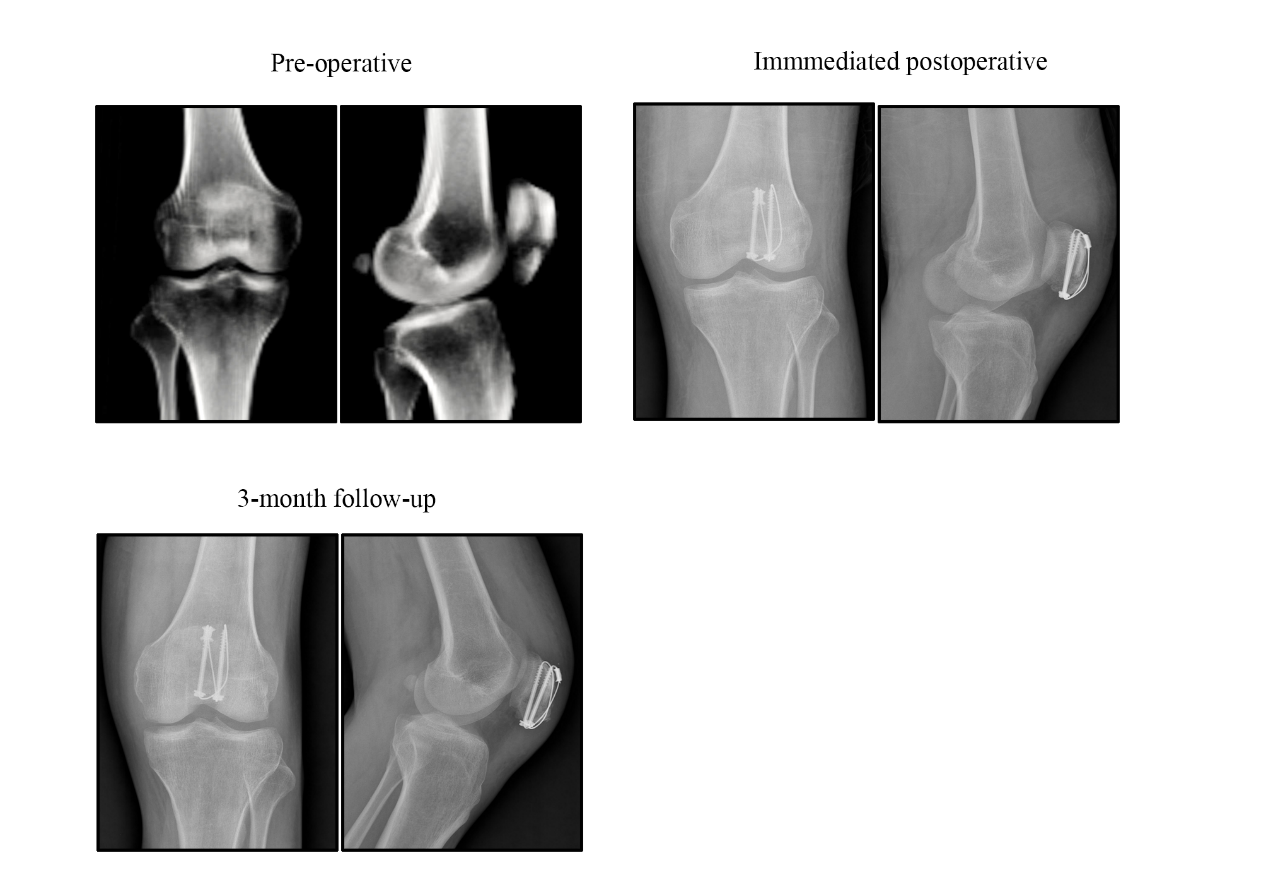


Figure S2. A typical case of the ATBW group. A. This is a 54-year-old male patient with IPFP. B. The patient underwent reconstruction of the IPFP through ATBW fixation. C. After a 3-month follow-up, the patella fracture healed well, and the injured knee has full range of motion.

Figure S3


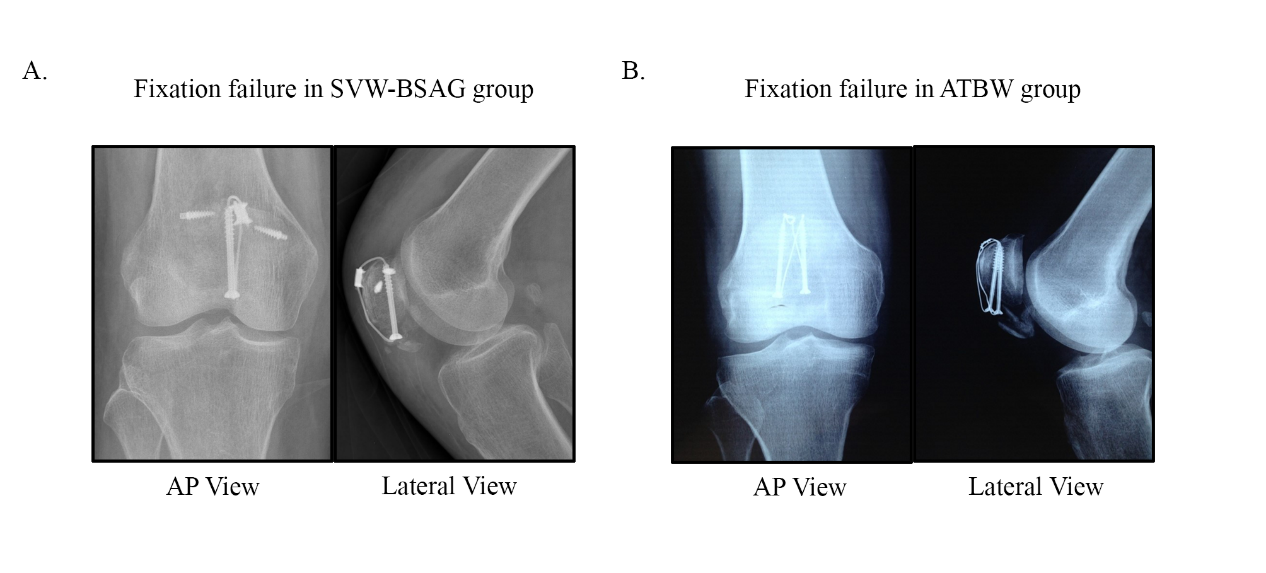


Figure S3. Fixation failure case in the two groups.

Table S1. Elements and nodes of the three models.

|  | ATBW Model | SVW Model | SVW-BSAG Model |
| --- | --- | --- | --- |
| Elements | 519200 | 510000 | 534506 |
| Nodes | 749001 | 729214 | 777757 |
